# Analyzing arguments on tobacco tax increases. Focus on French parliamentary questions and responses, 2000–2020

**DOI:** 10.18332/tid/175618

**Published:** 2024-01-08

**Authors:** François Topart, Emmanuelle Béguinot, Karine Gallopel-Morvan

**Affiliations:** 1 Comité National Contre le Tabagisme (CNCT), Paris, France; 2 University Rennes, EHESP, CNRS, Inserm, Arènes - UMR 6051, RSMS - U 1309, Rennes, France

**Keywords:** tobacco industry, tobacco taxation, lobbying, parliamentary documents, France

## Abstract

**INTRODUCTION:**

Tax increases are the most effective but still the least-used tobacco control measure. The tobacco industry (TI) employs lobbying strategies to oppose the implementation of tax policies on its products. Over the past two decades, French tobacco tax policies have been characterized by a relative inconsistency. This research aims to understand why, by analyzing the arguments of French policymakers (MPs and government) between 2000 and 2020 in favor or against tax increases.

**METHODS:**

To capture parliamentary debates, we performed an advanced term search on the French National Assembly website, using the keyword ‘tobacco’. The search returned 5126 available documents out of which 1106 (12.6%, 645 questions, 461 responses) covered price and taxation and were included. They were analyzed using descriptive statistics and thematic content analysis (NVivo) and were compared, when relevant, to arguments raised in the international literature on TI lobbying against taxation increases.

**RESULTS:**

We found 3176 arguments on tobacco taxation: 77.2% were against tobacco tax increases and 22.7% were in favor of tax policies. Arguments varied depending on the source: 92.4% of MPs’ arguments were against tax increases, while 52.1% of arguments from government responses were in favor. The anti-tax arguments were similar to those identified in the international literature that singled out negative economic and social consequences (illicit trade, penalizing tobacconists). Other arguments that were more specific to the French context, highlighted the key economic and social role played by tobacconists in France. Pro-tax arguments highlighted the health, economic and social benefits of tax policies.

**CONCLUSIONS:**

This is the first French tobacco research on parliamentary documents, although Parliament is a place of direct TI lobbying. It will enable public health actors to better understand the arguments used by the TI in order to counter them in front of MPs, and to better monitor debates in Parliament.

## INTRODUCTION

Tobacco consumption is a major factor in the development of non-communicable diseases such as cancer or cardiovascular diseases^1^. Every year, smoking is responsible for the premature death of more than 8 million people worldwide and 75000 in France^2,3^. In France, despite a significant decrease between 2016 and 2019, smoking prevalence remains high; in 2022, 24.5% of those aged 18–75 years, were daily smokers^4^.

To address the tobacco epidemic, Article 6 of the WHO Framework Convention on Tobacco Control (FCTC) recommends to implement ‘price and tax measures [that] are an effective and important means of reducing tobacco consumption’^5^. Significant and repeated tax increases make tobacco less affordable, preventing young people from starting smoking and increasing the number of quit attempts among smokers^6,7^. Tobacco taxation is also effective among low-income populations who are more price-sensitive, and generates tax revenues that can be earmarked for tobacco control policies^8^.

Because tobacco taxation decreases public demand and reduces manufacturers’ profit margins^7^, the tobacco industry (TI) seeks to keep taxation on its products as low as possible^9,10^. The Policy Dystopia Model (PDM) develops a critical conceptual model of the TI political activity and a taxonomy of discursive and instrumental strategies, notably aimed at influencing taxation policies^11^. These discursive strategies produce an ‘alarmist narrative’, articulated around arguments asserting that ‘tax increases lead to illicit trade’; ‘they benefit undeserving groups’; ‘they are regressive and unfair, and penalize poor consumers’; ‘they lead to unanticipated costs for the economy, for society, for public authorities’; and ‘there is no proven link between increased tobacco taxation and decreased tobacco consumption’. Most of the literature dedicated to the TI’s lobbying against tax increases focuses on Anglosphere countries^12,13^. However, although the TI develops global lobbying strategies, it also adapts these strategies to local contexts^12,14^.

In the case of France, the TI arguments and lobbying in general remain under-researched despite that the Global Tobacco Index highlights a deterioration in the protection of French public policy from the TI’s influence^15^. Thus, although WHO FCTC obliges France to ‘protect [its] policies from […] interests of the tobacco industry’ (Article 5.3)^5^, public health authorities attribute the limited effectiveness of French tobacco control policies to tobacco sector lobbying^16^.

This gap is also paradoxical given that France has one of the highest smoking rates in the European Union^17^. France is also specific regarding tobacco sales: the legal sale is exclusively carried out by 23500 tobacconists that operate under the authority of the French Customs. This network is represented by the National Confederation of Tobacconists, which brings together 90% of all tobacconists in France^18^. This confederation maintains close ties with the TI^19,20^, from which it receives funding and for which it plays the role of a front group^16^. Given their status and territorial coverage, tobacconists share close ties with public policymakers^16^.

From 2000 to 2020, various periods alternated in France regarding tax rises (Table 1): high taxes increases favorable to public health (Periods 1 and 5), tax freezes or modest increases more favorable to the TI (Periods 2–4)^21-23^. In such a context, the aim of our research is to better understand the debates and arguments spread in Parliament on tobacco taxation, and thus to analyze whether the TI’s arguments identified in the literature are disseminated in a non-Anglosphere context, and whether the specific French context reveals emerging arguments, contributing to broadening the existing taxonomy of the TI’s arguments. We wish to clarify answers to the following questions:

**Table 1:** Change in cigarette pack price in France (2003–2021)

Period	*Date range*	*Context*	*Increase (€)*	*Increase (%)*	*Price of the best-selling pack (€)*	*Sales volumes (billions of units)*	*Variation (%)*
1	2003	First National Anti-Cancer	4.08	13.3	0.48	69648	-13.51
	2004	Program	0.92	22.5	5.00	54924	-21.14
2	2005	Tobacco tax moratorium	Stable		5.00	54801	-0.22
	2006				5.00	55772	1.77
3	2007	Slow and continuous price increase	0.13	2.60	5.13	54945	-1.48
	2008		0.17	3.30	5.30	53589	-2.47
	2009		0.05	0.90	5.35	54980	2.60
	2010		0.30	5.60	5.65	54797	-0.33
	2011		0.33	5.60	5.98	54108	-1.26
	2012		0.32	5.30	6.30	51456	-4.90
	2013		0.40	6.30	6.70	47527	-7.64
	2014		0.30	4.50	7.00	45014	-5.29
4	2015	Prices reached a 7€ plateau	Stable		7.00	45457	0.98
	2016				7.00	44926	-1.17
	2017				7.05	44261	-1.48
5	2018	National Tobacco Control Strategy (2018–2022) that set a price of 10€ for a cigarette pack	0.83	11.8	7.88	40232	-9.1
	2019		0.90	11.4	8.78	37207	-7.52
	2020		1.17	13.3	9.95	35817	-3.74

- What arguments are used in French parliamentary debates on tobacco taxation during the period studied (2000–2020) (in favor, against)?
- To what extent do these arguments overlap with those identified in the Anglosphere literature on the lobbying against tobacco tax increases (the PDM in particular)?
- How did the frequency of publication of parliamentary questions on tobacco taxation evolve during this period?
- What is the geographical origin of the MPs submitting the questions, and which ministries are targeted? We analyze this information to determine whether MPs from border departments show higher parliamentary activity than non-border MPs, in connection with the idea spread through the press that tax increases lead to cross-border trade to the detriment of tobacco retailers^24^. By focusing on the targeted ministries by MPs it is possible to determine whether they consider tobacco taxation as an economic or a health issue.

## METHODS

Following the qualitative method, we analyzed the publicly available documents of the National Assembly as it plays a crucial role as the place for debate on tobacco taxation in France. This is because the annual tobacco tax bill is drafted by the government and is submitted to the vote of the MPs who may amend, reject, and initiate the tax policy, or submit questions to challenge the government’s action. Thus, as the government has to publish its response within two months, questions can be a lobbying tool to influence the political debate^25^. All these documents will be used to analyze arguments around taxation policy, and to highlight if these arguments are close or not to those identified in the literature and in the PDM model^9,10^.

### Data collection

**Figure 1:**
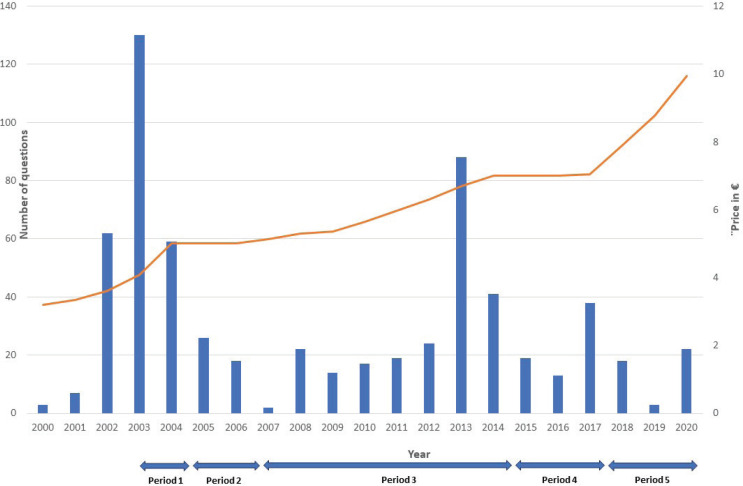
Number of MPs’ questions per year regarding tobacco price increases, and the 5 key periods of tobacco taxation policies in France, 2000–2020 (N=645)

Documents from parliamentary debates on tobacco taxation were collected using the advanced question search function on the French National Assembly website (https://www2.assemblee-nationale.fr). The timeframe studied was from January 2000 to December 2020, i.e. covering the five periods described in Figure 1 and Table 1. This timeframe has been retained because the year 2000 corresponds to the first significant tax increases implemented in France and to a shift in the Confederation’s strategy for lobbying public authorities^18^, while 2020 corresponds to the end of the fiscal policy initiated in 2017. A single search performed in 2021 with the keyword ‘tobacco’ resulted in 5216 documents. They were all read, and only questions that explicitly mentioned tobacco taxation were included, leading to a total of 645 questions. We read all the government responses to these 645 questions. Only responses mentioning tobacco taxation were kept, corresponding to a total of 461 responses, giving a total corpus of 1106 documents.

### Analysis

For each question, the following information was collected: date and frequency (the date used is the day of publication in the French Official Journal (*Journal Officiel*); the ministry to which the question is addressed; and the administrative division (*département*) that the MP represents. For each question, the MP’s department was coded in the National Institute of Geographical and Forestry Information software (*Institut national de l’information géographique et forestière, IGN*) to map the correlation between the geographical status of MPs and the number of questions they submit.

First, we used descriptive statistics to analyze the frequency of MPs’ questions through the 5 periods. Second, a thematic content analysis using NVivo 12 software (Lumivero company) was carried out to identify the arguments used in the questions and responses. It consists of ‘systematically identifying, grouping and, secondarily, examining the discursive themes addressed in a corpus’^26^. The coding process was the following: for arguments against tax increases identified in the literature and in the PDM, the coding grid was based on the previous classification. For arguments against tax not identified in the literature on lobbying against tobacco tax, and for arguments in favor of tax increases, an inductive analysis was used to fill in the grid. A researcher independently carried out the coding. When classification of arguments in the grid was unclear, the research team (three people) met to discuss the issue and reach agreement.

## RESULTS

### Geographical origin of MPs who ask questions and ministries targeted

**Figure 2:**
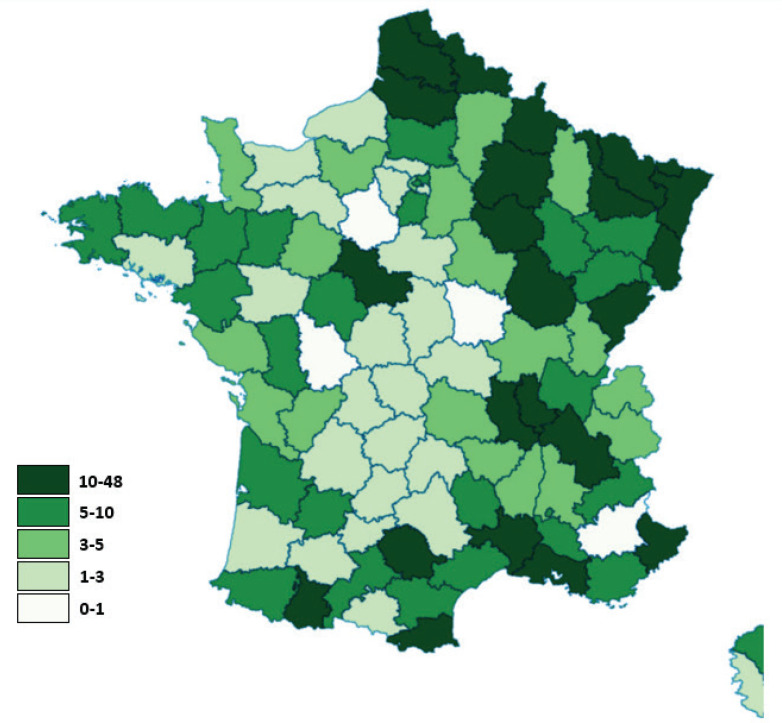
Amount of questions asked by MPs per department of France (2000–2020)

We analyzed whether border MPs submitted more questions than non-border ones, in connection with the idea that they might be likely to be against tax increases to protect border tobacconists. Over the period 2000–2020, published questions came from MPs representing 95 out of the 101 French administrative divisions (94.1%) (Figure 2). Of the 79 non-border department MPs, 74 submitted questions on tobacco taxation (93.7%). Of the 22 border-region department MPs, 21 engaged in parliamentary activity (95.5%). MPs from border departments asked relatively more questions than MPs from non-border departments: the 22 border-region departments represent 21.4% of all French departments and accounted for 39.4% of questions. On average, each department accounted for 6.3 questions (median= 4), 4.8 questions for non-border departments and 11.5 questions for border departments. MPs from departments bordering Belgium or Luxembourg asked 3.1 more questions than the average.

**Figure 3:**
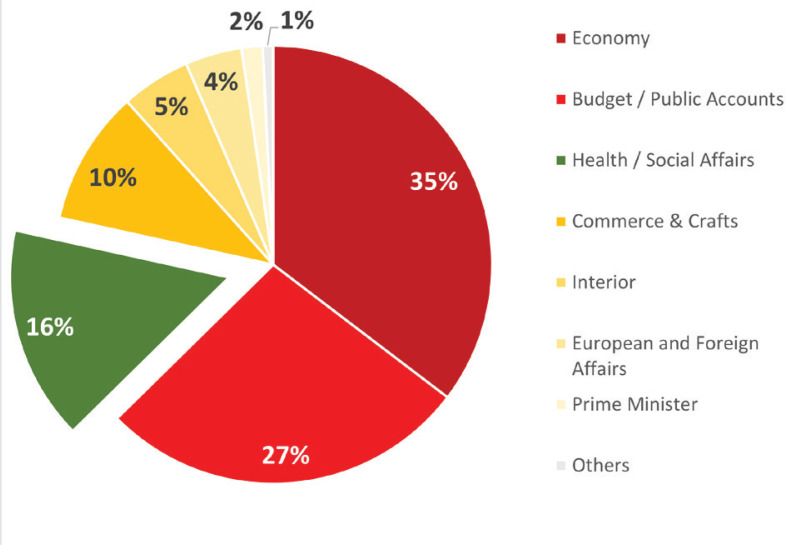
Ministries targeted by MPs’ questions (2000–2020)

The questions were categorized based on the ministry to which they were addressed (information specified in the published question) (Figure 3). Overall, the Ministry of the Economy was one of the most heavily targeted bodies by MPs (35%, n=228): 27% of the questions were submitted to the Ministry of Budget/Public Accounts (n=176) and 16% to the Ministry of Health/Social Affairs (n=102), with 10% submitted to the Ministries of Commerce, Crafts, and Small and Medium-sized Enterprise (n=64), 5% to the Ministry of the Interior (n=33), 4% to the Ministry of European and Foreign Affairs (n=27), and 1.1% to the Prime Ministers in power over the period studied (n=10).

**Figure 4:**
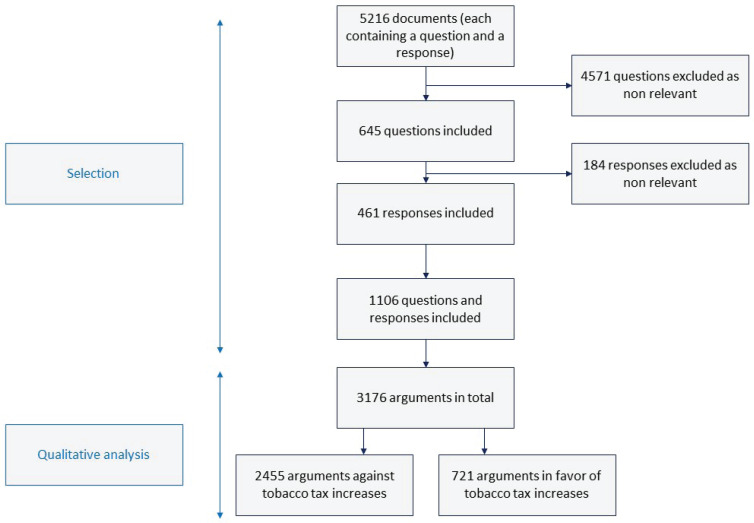
Flowchart for the selection of parliamentary documents and argument analysis process

### Frequency of questions during the period

For the period 2000–2020, there were three peaks in the frequency of questions asked by MPs, gathering 65% of the total amount of the 645 questions (Figure 1). The first peak, in 2003–2004, corresponds to a phase of substantial tax increases implemented in France and discussed from 2002 (Period 1 of the taxation trends described in Table 1). The second peak, in 2013–2014, corresponds to the discussions on the first National Tobacco Reduction Program (Period 3), which initially provided for the implementation of tax increases. The third peak, in 2017, corresponds to discussions around the strong tax increases that characterize Period 5. The period 2005–2012 was marked by little parliamentary activity on tobacco-related issues and corresponds to the implementation of a tobacco tax freeze (Period 2) and small but continuous tax increases (Period 3), which the French High Council for Public Health (*Haut Conseil de la santé publique*) described as insufficient to reduce tobacco consumption^27^.

### Overview of the arguments

In the 1106 parliamentary documents included for analysis, we identified 3176 arguments (Tables 2 and 3): 2455 against tax increases (77.2%) and 721 in favor (22.7%). A large majority of the arguments against were found in questions (n=1938; 78.9%), whereas a large majority of arguments in favor were found in responses (n=563; 78%). Most of the arguments identified in questions were against tax increases (92.4%), whereas the arguments put forward in responses were mainly in favor of tax policies (52.1%).

**Table 2:** Arguments against tax increases on tobacco products (2000–2020)

*Arguments*	*Occurrence of questions*	*Occurrence of responses*
**A) Tax increases result in unanticipated costs to the economy and society**	**1188**	**191**
**Tax increases have costs to the economy and society**	**888**	**43**
Tax increases lead to costs for tobacconists	425	37
Tax increases lead to costs for the tobacco sector (excluding tobacconists)	47	4
Tax increases have costs for the state and the community	62	0
Tax increases favor the parallel market and illicit trade	333	2
Tax increases result in a climate of insecurity	21	0
**Price differentials between France and EU or border countries have costs to the economy and society**	**280**	**148**
Price differentials favor the parallel market and illicit trade	141	104
Price differentials result in costs for tobacconists	99	39
Price differentials result in costs for the state and the community	26	0
The closure of the borders during the COVID-19 pandemic highlighted the scale of parallel markets	13	5
Price differentials result in costs for the tobacco sector (excluding tobacconists)	1	0
**The lack of tax harmonization in Europe has costs to the economy and society**	**20**	**0**
The lack of tax harmonization is detrimental to tobacconists	11	0
The lack of tax harmonization favors the parallel market and illicit trade	9	0
**B) Tobacconists play a vital economic and social role**	**468**	**251**
Tobacconists represent a profession that has great social utility, playing a major role in rural areas	252	186
Tobacconists offer a wide range of non-tobacco services, including public services	105	52
The huge number of tobacconists	82	8
Tobacconists are in favor of public health	14	1
Tobacconists are a symbol of conviviality and French tradition	10	3
The tobacconists’ monopoly enables better control of tobacco sales	5	1
**C) Tax increases are a regressive measure because they are socially unfair or abusive**	**51**	**0**
Tax increases penalize the buying power	17	0
Tax increases are unfair to tobacconists	12	0
Tax increases are brutal and exaggerated	11	0
Tax increases are thoughtless	5	0
Public health is a pretext for increasing taxes on tobacco	4	0
Tax increases are a demagogical measure	1	0
Experts consider tax increases unwise	1	0
**D) Tax increases benefit undeserving groups**	**34**	**0**
Tax increases benefit delinquency and criminal groups	24	0
Tax increases benefit foreign economies and actors	8	0
Tax increases are only passed in order to fill the state’s coffers	2	0
**E) Tax increases are ineffective or partially ineffective**	**129**	**57**
Tax increases are not effective	51	0
Tax increases have limited effectiveness	38	57
Tax increases undermine public health objectives	29	0
Tax increases are only effective if implemented along with complementary measures	11	0
**F) Increases on tobacco products should be limited**	**68**	**18**
It is necessary to limit, level out or space out tax increases	13	16
It is necessary to implement a tax freeze on tobacco products	22	2
It is necessary to maintain certain preferential tax regimes (airports, Corsica)	13	0
It is necessary to implement tax abatements/new tax-free zones	12	0
Other measures are preferable	8	0
**TOTAL**	**1938**	**517**

**Table 3:** Arguments in favor of tax increases on tobacco products (2000–2020)

*Arguments*	*Occurrences of questions*	*Occurrences of responses*
**A) Tax increases are beneficial for public health**	**139**	**429**
Tax increases are decided with a health objective (especially the health of young people)	91	279
Tax increases are an essential tool for tobacco control	0	18
**Tax increases are an effective tool in the fight against tobacco use**	**48**	**132**
Tax increases reduce tobacco consumption	18	46
Tax increases reduce tobacco sales	17	61
The effectiveness of tax increases is established	13	25
**B) Tax increases are good for the economy and society**	**7**	**78**
Tax increases are beneficial to the economy and society	7	7
**Tax increases are beneficial to the tobacco industry**	**0**	**71**
Tobacco remuneration has increased for tobacconists	0	45
Tobacco remuneration has increased for frontier tobacconists	0	8
Tobacco tax increases result in increased revenue for tobacconists	0	17
Tax increases do not penalize manufacturers	0	1
**C) Taxes need to be raised, a moratorium on taxation should not be granted**	**12**	**56**
**TOTAL**	**158**	**563**

### Arguments against tax increases

We identified five categories of arguments against tax increases in the parliamentary documents (Table 2) previously identified in the PDM: Tax increases result in unanticipated costs to the economy and society; Tax increases are ineffective or only partially ineffective; Tax increases on tobacco products should be limited; Tax increases are a regressive measure because they are socially unfair or abusive; and Tax increases benefit undeserving groups. We also identified an emerging category of argument, specific to France, highlighting the vital economic and social role of tobacconists that could be disrupted by tobacco taxes. These six arguments are presented in order of their recurrence in the questions and responses.

*Tax increases result in unanticipated costs to the economy and society*

This category is divided into three subcategories: 1) Tax increases have costs to the economy and society [Questions (Q): n=888; Responses (R): n=43]; 2) Price differences between France and EU or border countries have costs to the economy and society (Q: n=280; R: n=148); and 3) The lack of tax harmonization in Europe has a cost to the economy and society (Q: n=20; R: n=0).

Subcategory ‘1’ is further subdivided into arguments substantiating the costs associated with tax increases: tax increases have costs for tobacconists (Q: n=425; R: n=37), tax increases have costs for the tobacco sector (excluding tobacconists) (Q: n=47; R: n=4), tax increases have costs for the state and the community (Q: n=62; R: n=0), tax increases favor the parallel market and illicit trade (Q: n=333; R: n=2), and tax increases result in a climate of insecurity’ (Q: n=21; R: n=0).

Subcategory ‘2’ is subdivided into arguments substantiating the costs associated with price differentials: price differentials encourage the parallel market and illicit trade (Q: n=141; R: n=104), price differentials result in costs for tobacconists (Q: n=99; R: n=39), price differentials result in costs for the state and the community (Q: n=26; R: n=0), the border closures during the COVID-19 pandemic highlighted the scale of parallel markets (Q: n=13; R: n=5), and price differentials have costs for the tobacco sector (excluding tobacconists) (Q: n=1; R: n=0).

Subcategory ‘3’ is subdivided into arguments substantiating the costs associated with the lack of tax harmonization between France and border countries: the lack of tax harmonization is detrimental to tobacconists (Q: n=11; R: n=0), and the lack of tax harmonization favors the parallel market and illicit trade (Q: n=9; R: n=0).

*Tobacconists play an important role: arguments specific to France*

This category gathers arguments portraying tobacconists in a highly positive light, describing them as vital actors of French society. This category is subdivided into the following sub-arguments: tobacconists represent a profession that has great social utility, playing a major role in rural areas (Q: n=252; R: n=186); tobacconists offer a wide range of non-tobacco services, including public services (Q: n=105; R: n=52); the huge number of tobacconists (Q: n=82; R: n=8); tobacconists are in favor of public health (Q: n=14; R: n=1); tobacconists are a symbol of conviviality and French tradition (Q: n=10; R: n=3); and the tobacconists’ monopoly enables a better control of tobacco sales (Q: n=5; R: n=1).

*Tax increases are (partially) ineffective*

This category is subdivided into four sub-arguments: tax increases are not effective (Q: n=51; R: n=0), tax increases have limited effectiveness (Q: n=38; R: n=57), tax increases undermine public health (Q: n=29; R: n=0), and tax increases are only effective if implemented along with complementary measures (Q: n=11; R: n=0).

*Tax increases on tobacco products should be limited*

This category is subdivided into five sub-arguments: it is necessary to limit, level out or space out tax increases (Q: n=13; R: n=16); it is necessary to implement a tax freeze on tobacco products (Q: n=22; R: n=2); it is necessary to maintain certain preferential tax regimes (airports, Corsica) (Q: n=13; R: n=0); it is necessary to implement tax abatements/new tax-free zones (Q: n=12; R: n=0); and other measures are preferable (Q: n=8; R: n=0).

*Tax increases are regressive*

These argue that tax increases are unfair, brutal, and unprepared. Only identified in questions, this category is subdivided into sub-arguments: tax increases penalize the purchasing power (n=17), tax increases are unfair to tobacconists (n=12), tax increases are brutal and exaggerated (n=11), tax increases are thoughtless (n=5), public health is a pretext for increasing taxes on tobacco (n=4), tax increases are a demagogical measure (n=1), and experts consider tax increases unwise (n=1).

*Tax increases benefit undeserving groups*

This category is subdivided into three sub-arguments that were only found in questions: tax increases benefit delinquency and criminal groups (n=24), tax increases benefit foreign economies and actors (n=8), tax increases are only passed in order to enrich the state (n=2).

To summarize the arguments against taxation, the issue of the cost of tobacco taxation to the economy and society was particularly prevalent in the questions and responses, especially given the amount of arguments linking tobacco taxation to parallel markets. The emerging argument highlighting the key economic and social role played by tobacconists was also common in the documents. Other arguments, identified in the PDM, are rarely present in parliamentary documents, such as arguments stressing the regressive nature of tax increases, or the fact that they benefit undeserving groups.

### Arguments in favor of tax increases

We identified three categories of arguments in favor of tax increases in the parliamentary documents not included in the literature. They are presented below and detailed in Table 3.

*Tax increases are beneficial for public health*

This category is subdivided into three sub-arguments: tax increases are decided with a health objective (especially the health of young people) (Q: n=91; R: n=279), tax increases are an essential tool for tobacco control (Q: n=0; R: n=18), and tax increases are an effective tool for tobacco control (Q: n=48; R: n=132). This sub-argument groups three statements: tax increases reduce tobacco consumption (Q: n=18; R: n=46), tax increases reduce tobacco sales (Q: n=17; R: n=61), and tax increases are effective (Q: n=13; R: n=25).

*Tax increases are beneficial for the economy and society*

This category is subdivided into two sub-arguments: tax increases are beneficial to the economy and society (Q: n=7; R: n=7), and tax increases are beneficial to the TI (Q: n=0; R: n=71). This sub-argument groups four statements: tobacco remuneration has increased for tobacconists (n=45), tobacco remuneration has increased for frontier tobacconists (n=8), tobacco tax increases result in increased revenue for tobacconists (n=17), and tax increases do not penalize manufacturers (n=1).

*Taxes need to be raised*

This category gathers arguments asserting that taxes should be increased and that no tax abatements or moratoriums should be granted (Q: n=12; R: n=56).

To summarize the pro-tax arguments, those pointing to the public health benefits of taxation were frequent in government’s responses defending its taxation policy, but rare in MPs’ questions. Arguments pointing out that tobacco tax policies result in benefits for the economy and society were rare in responses, and absent from questions.

## DISCUSSION

This research is the first French study that examines arguments disseminated in parliamentary documents in general, and the first that examines arguments on tobacco taxation arguments.

First, it reveals that in France, MPs from border areas tend to submit more questions on tobacco taxation than non-border MPs. As one of the major arguments against taxation increases is linked with cross-border and illicit trade, this result could indicate that tobacconists of border departments are close to MPs and pressure them to disseminate arguments in parliamentary debates, as tobacconists do through the general press^24^.

Second, more MPS’ questions were addressed to the Ministry of the Economy/Budget than to the Ministry of Health. This result could indicate that MPs grasp tobacco taxation more as an economic paradigm than a public health one, and that they spread opposed arguments to ministries that could be more sensitive to economic arguments than health arguments.

Third, three peaks of questions, mostly opposed to tax increases, were identified over the 2000–2020 period, corresponding to key moments for tobacco taxation in France. This could indicate an intensification of direct lobbying of the TI and tobacconists toward MPs that could be used by these actors to spread anti-taxation arguments in parliamentary debates.

Fourth, anti-taxation arguments were predominant in the period studied. Most of them were included in MPs’ questions, whereas government’s responses were more balanced. As the role of MPs is to represent constituents, it could be assumed that they are more likely to relay their concerns, including those from tobacconists. This predominance of anti-taxation arguments is also identified in the general press^24^. It could indicate the existence of direct lobbying by economic actors aimed at spreading anti-taxation arguments through MPs. This could be consistent with observations by French public health authorities, who stress the issue of the TI’s influence and MPs’ attempts to weaken tobacco control legislation^16,28^.

The arguments against tobacco taxation were similar to the arguments identified in the PDM, particularly those relating to unanticipated costs to the economy and society, or to illicit trade. Other arguments identified in the PDM (e.g. tax increases are unfair or benefit undeserving actors) also emerged, but to a less extent.

Our analyses also found specific French arguments in connection with tobacconists. They are portrayed as key actors in social and economic life, particularly in rural areas, and as the main victims of tax policies. This representation is consistent with the tobacconists Confederation’s strategy, which presents tobacconists as ‘retailers who provide social links and ensure the territorial continuity of public services’^18^. This could suggest that these actors are presented to some MPs as a part of the traditional French economy and must be defended against effective tobacco policies. This French emerging argument is quite close to the PDM classification and reinforces a dystopian narrative, penalizing ‘a wide range of stakeholders, damaging the economy and society as a whole’^11^.

Finally, our research also revealed arguments in favor of tax increases (health benefits) that have not been included in the Anglosphere literature^11,12^. It may reveal that arguments disseminated by public health actors reach MPs, but to a far less extent than arguments against taxation increases. This could also suggest a tobacco denormalization process among some policymakers.

This research makes several contributions to the existing literature. Despite tax increases are a particularly effective tool for reducing tobacco consumption^6^, our study reveals that debates in Parliament are strongly influenced by anti-taxation arguments, and that the French anti-taxation discourse is structured around the penalization of tobacconists. This confirms that the PDM provides an appropriate analysis framework, but also requires ‘flexibility to capture context-specific strategies’^14^.

Concerning contributions for public health, our research underlines the need to monitor parliamentary debates to analyze anti-taxation arguments and combat them through direct lobbying to MPs and through the general press. As the arguments disseminated in parliamentary debates are very close to those conveyed by the TI and tobacconists, it is necessary to take better account and implement Article 5.3 of the WHO FCTC in France^29^. Our research also enables public health actors to counter the positive narrative associated with French tobacconists and highlights the need for public health actors to map MPs to identify potential allies and opponents to tobacco taxes. It could also help to develop a discursive strategy that competes with the TI’s narrative, by highlighting the various positive impacts of tax policies^30^, not just health benefits, but also economic and societal ones.

### Limitations

Despite these important results for public health, our research carries limitations. First, as the data collection was done manually by a single researcher, some relevant documents could have been missed. However, given the quantity of documents collected, the results would not have been significantly modified. Second, inherent to qualitative research, analysis is exposed to subjective bias^26^. We tried to limit this bias by working among the team to find a consensus when doubts emerged on classification of some arguments. Third, since this study focuses on the specific French context, the generalizability of our findings to other countries is limited, especially for those without a monopoly on tobacco sales, or less exposed to cross-border trade. Fourth, if analyzing parliamentary debates is relevant to highlight lobbying arguments^31-35^, they must be completed by other analyses, including other parliamentary documents (amendments, minutes of committee debates or plenary sessions), arguments disseminated through the trade press, general press, and interviews of MPs, to better understand how they set up their decision in favor or against tobacco taxation.

## CONCLUSIONS

Although tobacco tax increases are an effective tool for reducing smoking prevalence, most arguments circulating in French parliamentary debates were against this measure and similar to those identified in Anglosphere literature. This opposition could indicate that the TI develops direct lobbying strategies towards MPs, aimed at spreading arguments to block tax increases.

Given the Parliament’s key role in setting tobacco tax policy, our results have three implications for public health actors: 1) the importance to monitor parliamentary debates and map MPs to identify key interlocutors, potential allies and opponents to tax policies; 2) the need to build a counter-argument to the one developed by the TI and to build a positive narrative around tax increases, highlighting the associated benefits (health, tax revenues, reduced inequalities); and 3) the importance of conducting further research on complementary parliamentary documents (amendments, minutes of committee and plenary debates).

## Data Availability

The data supporting this research are available from the authors on reasonable request.
